# The heterogeneity of astrocytes in glaucoma

**DOI:** 10.3389/fnana.2022.995369

**Published:** 2022-11-17

**Authors:** Yunjing Tang, Yongjiang Chen, Danian Chen

**Affiliations:** ^1^Research Laboratory of Ophthalmology and Vision Sciences, State Key Laboratory of Biotherapy, West China Hospital, Sichuan University, Chengdu, China; ^2^Department of Ophthalmology, West China Hospital, Sichuan University, Chengdu, China; ^3^The School of Optometry and Vision Science, University of Waterloo, Waterloo, ON, Canada

**Keywords:** astrocytes, heterogeneity, retinal ganglion cells, glaucoma, optic nerve head, intraocular pressure, inflammation, mitochondrial dysfunction

## Abstract

Glaucoma is a leading cause of blindness with progressive degeneration of retinal ganglion cells. Aging and increased intraocular pressure (IOP) are major risk factors. Lowering IOP does not always stop the disease progression. Alternative ways of protecting the optic nerve are intensively studied in glaucoma. Astrocytes are macroglia residing in the retina, optic nerve head (ONH), and visual brain, which keep neuronal homeostasis, regulate neuronal activities and are part of the immune responses to the retina and brain insults. In this brief review, we discuss the activation and heterogeneity of astrocytes in the retina, optic nerve head, and visual brain of glaucoma patients and animal models. We also discuss some recent transgenic and gene knockout studies using glaucoma mouse models to clarify the role of astrocytes in the pathogenesis of glaucoma. Astrocytes are heterogeneous and play crucial roles in the pathogenesis of glaucoma, especially in the process of neuroinflammation and mitochondrial dysfunction. In astrocytes, overexpression of Stat3 or knockdown of IκKβ/p65, caspase-8, and mitochondrial uncoupling proteins (Ucp2) can reduce ganglion cell loss in glaucoma mouse models. Based on these studies, therapeutic strategies targeting the heterogeneity of reactive astrocytes by enhancing their beneficial reactivity or suppressing their detrimental reactivity are alternative options for glaucoma treatment in the future.

## Introduction

Glaucoma is a leading cause of blindness, characterized by progressive degeneration of retinal ganglion cells (RGCs) and optic nerve head (ONH) remodeling (also called optic disc cupping). There were about 76 million glaucoma patients between 40 and 80 years old in 2020, which will likely increase to 112 million in 2040 (Tham et al., 2014). Glaucoma can be divided into open-angle and angle-closure glaucoma according to the anterior chamber angle. Glaucoma can also be classified as primary or secondary glaucoma (Jonas et al., 2017; Simcoe et al., 2020). Glaucoma is generally diagnosed based on optic disc cupping, high intraocular pressure (IOP), and visual field defects. Treatment to lower IOP is based on topical drugs, laser therapy, and surgical intervention if other therapeutic modalities fail to prevent progression (Jonas et al., 2017). However, some patients with controlled IOP still experience further optic nerve (ON) damage and vision loss. The sensitivity to IOP, progressive apoptosis of RGCs, thinning of the retinal nerve fiber layer (NFL), and cupping of the optic disc or ONH are considered hallmarks of glaucoma (Jonas et al., 2017; Sun et al., 2017).

The ONH is located nasally to the macula and allows the exit of RGC axons from the eye. The main ONH component is the lamina cribrosa (LC). Increased IOP can cause stress and strain on the lamina cribrosa, resulting in remodeling of this structure, thus blocking anterograde and retrograde axonal transport within the optic nerve fibers (Pease et al., 2000; Quigley et al., 2000). In most cases, the remodeling appears regional, with a preference for the superior and inferior portions of the ONH (Quigley and Green, 1979; Quigley and Addicks, 1981; Sommer et al., 1991; Tuulonen and Airaksinen, 1991). In addition, neuroinflammation and mitochondrial dysfunction are significant contributors to the pathogenesis of glaucoma (Ahmed et al., 2004; Yang et al., 2007; Williams et al., 2017; Tribble et al., 2021).

Glaucomatous loss of RGCs is often accompanied by changes in ocular glial cells, including Müller glia, astrocytes, and microglia. Astrocytes are especially important for glaucoma as they have long-lasting responses that broadly impact on RGCs in glaucoma. Astrocytes in the visual brain (Yucel and Gupta, 2015), such as the visual cortex, lateral geniculate nucleus (LGN), and superior colliculi (SCs), also play roles in the pathogenesis of glaucoma (Shimazawa et al., 2012). We will discuss the heterogeneity and major roles of astrocytes in the normal retina, ONH, and visual brain and major changes in glaucoma patients and animal models. The unique role of astrocytes in glaucoma pathogenesis is revealed in several transgenic or gene knockout studies. Understanding the crucial roles of astrocytes is essential for the early detection and development of novel glaucoma treatments to augment conventional IOP-lowering treatments.

## Searching Strategies

A systematic search of the PubMed database was conducted up to October 2022 to prepare and revise this brief review. Articles dealing with the astrocytes and pathogenesis of glaucoma were carefully selected and reviewed. The search terms used included word combinations such as “glaucoma and glia” (653 results), “glaucoma and astrocytes” (474 results); “ocular hypertension and astrocytes” (317 results); “optic nerve head and astrocytes” (431 results); “ocular neurodegeneration and astrocytes” (56 results). All titles and abstracts were then attentively read, and if the subject was compatible with our article, the article was reviewed in detail.

## The Heterogeneity and Functions of Astrocytes in The Visual Brain, ONH, and Retina

### Heterogeneity of astrocytes

#### Heterogeneity of visual brain astrocytes

**Figure 1:**
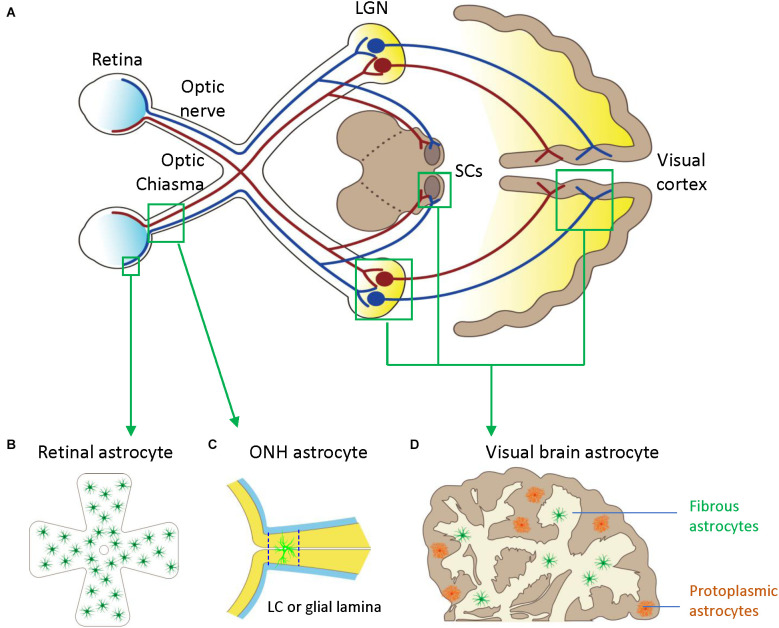
Astrocytes in the visual pathway may be related to the pathogenesis of glaucoma. **(A)** The schematic diagram showing the visual pathway, including retina, optic nerve, optic chiasma, LGN, SCs, and visual cortex. **(B)** Retinal astrocytes mainly reside in the nerve fiber layer (NFL) and orient in a monolayer surrounding RGC axons and blood vessels. **(C)** Optic nerve head (ONH) astrocyte processes are oriented perpendicularly to RGC axons, separating them into bundles. The two dash lines label the position of LC (human) or glia lamina (rat or mouse). **(D)** Visual brain astrocytes in LGN, SCs, and visual cortex, have two types including protoplasmic astrocytes of gray matter and fibrous astrocytes of white matter. LGN, lateral geniculate nucleus; LC, lamina cribrosa; NFL, the nerve fiber layer; ONH, optic nerve head; RGC, retinal ganglion cells; SCs, superior colliculi.

Astrocytes in the visual brain, ONH, and retina may participate in the pathogenesis of glaucoma (Figure 1). Both astrocytes and neurons originate from neural progenitor cells (NPCs) in the central nervous system (CNS). In the embryonic brain, NPCs generate neurons first and astrocytes later (Dinh Duong et al., 2019). Cell fate switch from neurons to astrocytes is crucial for matching numbers in each cell lineage (Wang et al., 2016). Astrocytes and neurons are represented in a ratio of 1:3 in the cortex of rats and mice and about 3:2 in the human cortex (Bass et al., 1971). Astrocytes spread the whole CNS in a contiguous and non-overlapping manner, and there are no CNS regions without astrocytes or closely related cells (Ogata and Kosaka, 2002; Bushong et al., 2004). Astrocytes have regional and morphological heterogeneity in the brain. At least there are two types of CNS astrocytes, protoplasmic astrocytes of gray matter and fibrous astrocytes of white matter; both make extensive contact with blood vessels and form gap junctions between distal processes of neighboring astrocytes (Sofroniew and Vinters, 2010).

#### Heterogeneity of ONH astrocytes

During the development, the mitotic precursors of astrocytes in the ONH come from the sub-ependymal germinal layer of the brain (Vecino et al., 2016). Human ONH has four regions: surface nerve fiber layer (NFL), pre-laminar, lamina cribrosa (LC), and post-laminar myelinated optic nerve. LC is a collagenous sieve-like plate consisting of astrocytes and LC cells. The rodent ONH does not contain a collagenous LC but a dense meshwork of astrocytes (glial lamina) that enwrap RGC axons and organize them into bundles. Astrocytes are the major glial cell type in the non-myelinated ONH in most mammals, including humans and mice. In the human ONH, astrocytes have regional and molecular heterogeneity suggesting differential regional responses to glaucoma (Ye and Hernandez, 1995). There are at least three subtypes of astrocytes, including type 1A, type 1B, and type 2. Type 1 astrocytes are in the unmyelinated LC and prelaminar region of the ONH, and type 2 astrocytes are in the myelinated post-laminar part of the ONH. Type 1A astrocytes are glial fibrillary acidic protein (GFAP) positive but neural cell adhesion molecule (NCAM) negative. Type 1B astrocytes, the major astrocyte subtype in the ONH, are GFAP^+^ and NCAM^+^ (Ye and Hernandez, 1995; Kobayashi et al., 1997).

In rodent ONH, there is only one defined astrocyte type (the fibrous astrocyte of white matter). Individual astrocytes are relatively large and usually span at least half the diameter of the nerve. They overlap extensively, which is different from non-overlapping cortex astrocytes. Their processes are oriented perpendicularly to RGC axons, separating them into bundles (Sun et al., 2009; Wang et al., 2017). The cytoplasm of rat ONH astrocytes is highly electron-dense and uniform throughout the entire cell processes, which have massive cytoskeletal strengthening of longitudinal massed filaments and tubules. As such, these astrocytes are called fortified astrocytes (Dai C. et al., 2012).

#### Heterogeneity of retinal astrocytes

ONH astrocytes migrate to the retina during the development and spread across the NFL (Hollander et al., 1991; Tao and Zhang, 2014). Retinal astrocytes mainly reside in the NFL and orient in a monolayer surrounding RGC axons and blood vessels; the density of astrocytes correlates closely with the density of blood vessels (Stone and Dreher, 1987; Watanabe and Raff, 1988). Human retinal astrocytes can be morphologically divided into two major subgroups, elongated and stellate-shaped astrocytes. The processes of the elongated astrocytes form densely packed bundles along with the RGC axons in the NFL, while the stellate-shaped astrocytes form a honeycomb-shaped plexus in the ganglion cell layer (GCL; Watanabe and Raff, 1988; Ramírez et al., 1996).

GFAP (Eng et al., 1971), vimentin (Shaw et al., 1981; Björklund et al., 1984; Yamada et al., 1992), and nestin (Clarke et al., 1994) are the major astrocytic intermediate filaments and represent reliable markers of astrocytes (Vecino et al., 2016). Pax2 (Stanke et al., 2010) and Sox2 (Kelley et al., 2018; Lozano et al., 2019) are also useful nuclear markers of astrocytes in the optic nerve.

### Functions of astrocytes

**Figure 2:**
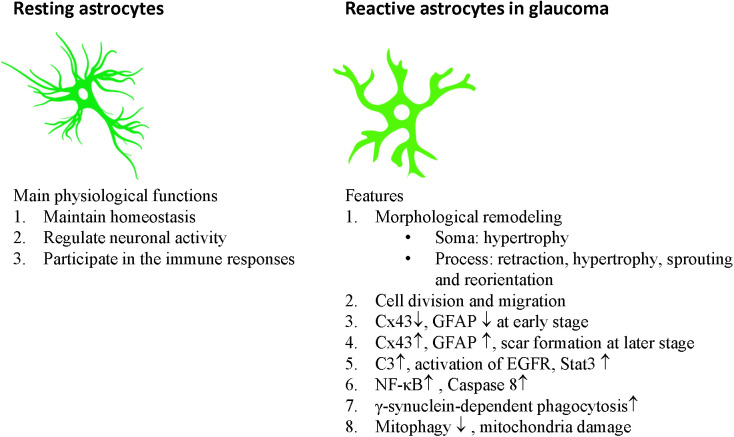
Main physiological functions of astrocytes and features of reactive astrocytes in glaucoma.

Astrocytes communicate with each other through gap junctions. Astrocytes have considerable plasticity and play important roles in balancing the normal retinal, ONH, and brain activity by maintaining homeostasis, regulating neuronal activity, and participating in immune responses (Table 1, Figure 2).

**Table 1:** Functions of astrocytes.

**Astrocytes functions**	**Major subjects of studies**	**References**
Maintain homeostasis	Boundaries and retinal blood barrier (BRB)	Vecino et al. (2016)
	Hypoxia and mechanical sensor	Choi et al. (2015) and Selvam et al. (2018)
	Nutritional supplements and water flow regulation	Fukuda et al. (2010)
Regulate neuronal activity	Synapse	Farhy-Tselnicker et al. (2017)
	Neurotransmitter	Cahoy et al. (2008)
	Neurotrophic factors (NF)	Dulz et al. (2020)
Participate in the immune responses	Reactive astrocytes	Liddelow and Barres (2017)
	A1 and A2 astrocytes	Liddelow et al. (2017)

#### Maintain homeostasis through multiple pathways

Retinal astrocytes provide structural support for retinal neurons by secreting multiple molecules (e.g., laminin, fibronectin, tenascin, and integrin) that form the extracellular matrix (ECM) and the inner limiting membrane (ILM), which is the boundary between the retina and the vitreous body (Jiang et al., 1994; Johnson et al., 2007; Vecino et al., 2016). Astrocytes send processes to retinal blood vessels and RGC axons and form glial boundaries between the retina and vascular vessels, part of the blood-retina barrier (BRB; Yao et al., 2014; Vecino et al., 2016; Fresta et al., 2020). Astrocytes participate in forming the inner BRB by forming tight junctions with Müller cells, microglia, endothelial cells, and pericytes (Holash and Stewart, 1993; Fresta et al., 2020).

CNS astrocytes are also necessary to maintain blood-brain barrier (BBB) integrity in the adult brain. BBB-regulating factors secreted by other cell types are insufficient to compensate for astrocyte loss (Heithoff et al., 2021; Morales et al., 2022). They also form gap junctions at astrocytic-endothelial interfaces and astrocytic-neuron interfaces (Ezan et al., 2012). Astrocytic gap junctions are built mainly of connexin-43 (Cx43), prevalent in astrocytes in the retina and ONH (Kerr et al., 2010, 2011). Gap junctions allow functional coordination among the members of the neurovascular unit, such as glial cells, endothelial cells, pericytes, and neurons (Rash et al., 2001).

Astrocytes act as hypoxia sensors and mechanosensors at these boundaries (Choi et al., 2015; Vecino et al., 2016). They can respond to hypoxia in the inner retina by expressing VEGF, thereby inducing the formation of the superficial vascular plexus (SVP) of the retina (Selvam et al., 2018). Thus, they are closely related to retinal vasculature development. Indeed, the astrocyte network acts as a template for the developing retinal vasculature, which also spreads out from the ONH (West et al., 2005). Astrocyte-specific *Pdgfa* gene knockout prevents astrocyte invasion into the retina and the development of retinal blood vessels (Fruttiger et al., 1996; Tao and Zhang, 2016). They can express fibronectin which binds VEGF to guide endothelial tip cell migration (Uemura et al., 2006). Endothelial filopodia protrusions closely align to astrocytes *via* adhesions formed between fibronectin and α5β1 integrin, and their genetic deletion impairs vascular migration (Stenzel et al., 2011).

Astrocytes can also respond to traumatic or glaucomatous injury by expressing mechanosensitive ion channels at ONH (Choi et al., 2015). They can respond to the translaminar pressure differentials in the post-laminar region of ONH by contacting pial septa filled with cerebrospinal fluid (Ren et al., 2010; Nguyen et al., 2011).

Astrocytes can provide nutritional supplements such as glucose and energy resources for surrounding neurons. Aquaporin channels (especially AQA-4) on astrocytes and Müller cells facilitate the bidirectional flow of free water, which allows them to regulate fluid volumes and modulate neuronal excitability (Fukuda et al., 2010).

#### Regulate multiple aspects of neuronal activity

Astrocytes regulate neuronal activity by modulating neuronal synapses, regulating neurotransmitter concentrations, and secreting neurotrophic factors in the CNS, including the retina. They are required to promote synaptic development and maturation and maintain synaptic stability and functionality in the CNS (Pfrieger and Barres, 1997; Mauch et al., 2001; Nägler et al., 2001; Ullian et al., 2001). The major pathways include thrombospondins (TSPs; Christopherson et al., 2005; Liauw et al., 2008), astrocyte-secreted glypican 4 (Gpc4; Farhy-Tselnicker et al., 2017), and apolipoprotein E (Apo E; Lorber et al., 2009) pathways.

Astrocytes also participate in synapse pruning. They continuously engulf both excitatory and inhibitory synapses through Megf10 and Mertk phagocytic pathways (Bishop et al., 2004; Chung et al., 2013). They can also eliminate synapses through complement-dependent pathways (Stevens et al., 2007). Astrocytes can regulate the concentration of neurotransmitters in the CNS. Astrocytes do not express vesicular glutamate transporters *in vivo* (Cahoy et al., 2008). However, they are rich in glutamine synthetase (GS), which transforms glutamate into glutamine and keeps the neuronal excitatory signals below harmful levels (Bush et al., 1999).

They take up excess extracellular potassium and glutamate, thus preventing neuronal excitotoxicity (Vecino et al., 2016). Astrocytes provide neuroprotection in the retina by secreting neurotrophic factors such as ciliary neurotrophic factor (CNTF; Dulz et al., 2020) and promote RGC axonal regeneration following injury (Cui et al., 1999; Leaver et al., 2006; Müller et al., 2007). Astrocytes are also a major source of glutathione, an antioxidant that fights against reactive oxygen species (ROS), which have been implicated in the pathogenesis of multiple neurodegenerative diseases (Chen et al., 2001; Shih et al., 2003; Guo et al., 2018).

#### Heterogeneous responses to local insults

Most glial cells are immune cells in the CNS. Reactive astrocytes are astrocytes that undergo morphological, molecular, and functional changes in response to disruption to the homeostatic state of the CNS and retina, leading to reactive astrogliosis (Escartin et al., 2021). The hallmark of reactive astrocytes is the upregulation of intermediate filaments, such as GFAP and vimentin (Liddelow et al., 2017). The reactive astrocytes form astrocytic scars, which separate regions of degeneration from healthy tissues, thus preventing degenerative effects and retaining the homeostasis of the extracellular environment (Bush et al., 1999; Anderson et al., 2016). However, ATP and glutamate released through gap junction from reactive astrocytes could also trigger neuronal loss (Orellana et al., 2011; Danesh-Meyer et al., 2012; Chen et al., 2015).

The response of astrocytes to local insults is very heterogeneous. While some evidence has suggested that reactive astrocytes can be polarized into the pro-inflammatory neurotoxic A1 state or anti-inflammatory neuroprotective A2 state (Zamanian et al., 2012; Liddelow et al., 2017), there are limitations of binary divisions of reactive astrocytes (Escartin et al., 2021). Indeed, astrocytes may exist as a continuum of A1 and A2 phenotypes shifting within a dynamic range of functional parameters rather than two distinct populations (Liddelow and Barres, 2017). Therefore, it might be difficult to distinguish the effects of one functional phenotype of astrocytes that takes part in the pathogenesis of glaucoma. Further studies are needed to determine astrocyte-based biomarkers and their impact on pathological hallmarks in relevant disease models, including glaucoma (Escartin et al., 2021).

## Heterogeneous Reactivity of Astrocyte in Glaucoma

In glaucoma patients, the typical loss of RGCs is not diffuse over the whole retina but arcuate, resulting in arcuate scotoma (Smith et al., 2017). RGCs loss in the glaucoma mouse model progresses in a fan-shaped sectorial pattern radiated from the ONH preceded by distal axon injury, axonal atrophy, dendritic remodeling, and soma shrinkage, suggesting the initial defect is axon damage at the ONH (Jakobs et al., 2005; Crish et al., 2010; Lye-Barthel et al., 2013). The slow, progressive degeneration of RGCs in glaucoma is a failure in both anterograde and retrograde axonal transport (Pease et al., 2000; Quigley et al., 2000; Ye et al., 2021). In addition to RGCs death and ONH remodeling, glaucomatous damage also extends to visual brain, including the superior colliculus (SC), lateral geniculate nucleus (LGN), and visual cortex (Dai Y. et al., 2012; Yucel and Gupta, 2015). Astrocytes in the retina, ONH, and visual brain may be involved in the pathogenesis of glaucoma (Figure 1). Reactive astrocytes are heterogeneous; they can change their gene expression profiles and induce neuroprotective or damaging influences at different locations and phases of glaucoma progression (Figure 2).

### Heterogeneous response of retinal astrocytes in glaucoma

**Figure 3:**
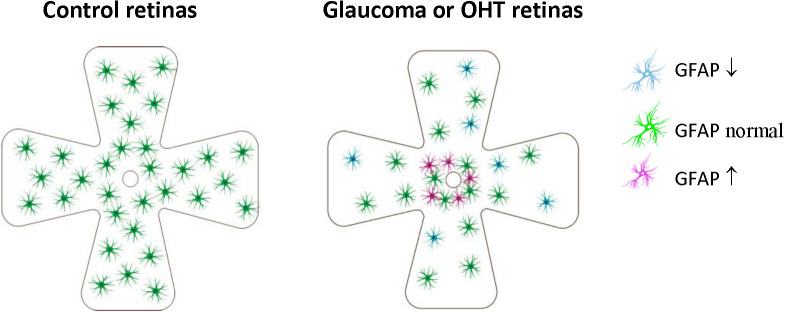
Retinal astrocyte activation in glaucoma is heterogeneous and is stage-dependent and spatially dependent. In the early stage of OHT/glaucoma, or the peripheral retina of late-stage glaucoma, cell density and GFAP expression of astrocytes are reduced. Astrocyte activation seems only to occur in some retinal areas of later-stage glaucoma, especially areas close to ONH. OHT, ocular hypertension; ONH, optic nerve.

Studies using SD rats with chronically elevated IOP induced by episcleral vein cauterization (EVC) found that retinal astrocytes lost GFAP expression 3 days after EVC, however their density increased steadily from 2 weeks to 6 months after EVC (Kanamori et al., 2005). Studies using SD rats or Swiss mice with laser-induced ocular hypertension (OHT) for 2–3 weeks found that astrocytes-occupied retinal area was reduced (Ramírez et al., 2010; Gallego et al., 2012; Table 2, Figure 3).

**Table 2:** Retinal astrocytes changes in glaucoma.

**Species, model, disease stage**	**Retinal astrocytes**	**References**
SD rats, 3 days of OHT	GFAP ↓	Kanamori et al. (2005)
SD rats, >2 weeks of OHT	GFAP ↑	Kanamori et al. (2005)
SD rats, 2–3 weeks of OHT	Decreased AROA	Ramírez et al. (2010) and Gallego et al. (2012)
Swiss mice, 2–3 weeks of OHT	Decreased AROA	Ramírez et al. (2010) and Gallego et al. (2012)
DBA/2J mice, 10–12months	Cell redistribution; variable GFAP expression	Formichella et al. (2014)
Human, late stage (postmortem)	Peripapillary regions: GFAP↑, Cx43 ↑ Mid-peripheral and peripheral retinas: GFAP GFAP ↓	Wang et al. (2002) and Kerr et al. (2011)

AROA, Area of the retina occupied by astrocytes; OHT, Ocular hypertension. Up arrow indicates increased expression of indicated proteins, down arrow indicates decreased expression of indicated proteins.

A study of GFAP-labeled astrocytes in human POAG retinas (donor age, 87.1 ± 6.9 years) found retinal astrocytes are spatial-dependent and heterogeneous. While the peripapillary regions (2 mm from the optic disc) had increased cell density and GFAP expression of astrocytes along large vessels, the mid-peripheral (6–8 mm from the optic disc) and peripheral retinas had reduced cell density and GFAP expression in most astrocytes, increased GFAP expression only found in some individual astrocytic bundles (Wang et al., 2002). Based on the available clinical records, there was no clear relationship between the severity of signs of glaucomatous damage and the changes in astrocytes (Wang et al., 2002). Another study of Cx43/GFAP-labeled astrocytes in human POAG retinas (donor age: 70 and 86 years) found increased connexin43 immunoreactivity in the peripapillary and mid-peripheral retina in association with glial activation (Kerr et al., 2011).

Similar spatial-dependent heterogeneity was found in DBA/2J mouse model of glaucoma, which suggested that astrocyte reactivity (increased density, soma, and GFAP expression) occur in micro-domains in the retina (Formichella et al., 2014). The mean density of astrocytes did not change, but astrocytes are re-distributed to produce lower or higher astrocyte density areas. Surprisingly, most astrocytes had decreased GFAP expression, while only a tiny group of astrocytes exhibited high levels of GFAP expression.

These results indicated that retinal astrocyte activation in glaucoma is heterogeneous, stage-dependent, and spatially dependent. In the early stage of OHT/glaucoma, or the peripheral retina of late-stage glaucoma, cell density and GFAP expression of astrocytes are reduced (Table 2, Figure 3). Astrocyte activation seems only to occur in some retinal areas of later-stage glaucoma. Astrocyte decline in the retina of glaucoma animals may contribute to RGCs loss (Formichella et al., 2014).

### Heterogeneous response of ONH astrocytes in glaucoma

**Figure 4:**
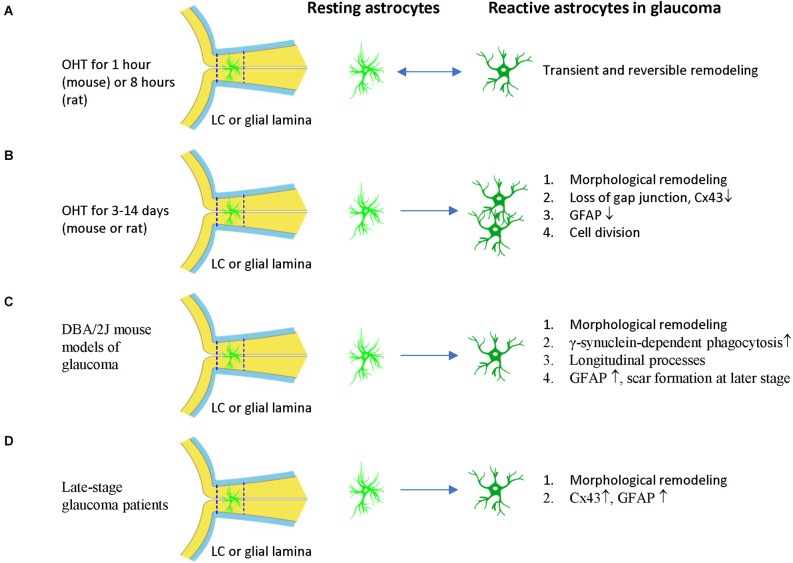
ONH astrocyte activation in glaucoma is heterogeneous and stage dependent. **(A)** Transient OHT induces reversible morphological remodeling of ONH astrocytes. **(B)** In the early stage of OHT, morphological remodeling persists, Cx43 and GFAP expression of ONH astrocytes are reduced. Cell division and cell number increased. **(C)** In DBA/2J mouse models of glaucoma, γ-synuclein-dependent phagocytosis increased, astrocyte spout longitudinal and GFAP^−^ processes. At late-stage, astrocytes can form scars. **(D)** In late stage of glaucoma patients, ONH astrocytes have morphological remodeling and increased expression of CX43 and GFAP. The two dash lines label the position of LC (human) or glia lamina (rat or mouse). LC, lamina cribrosa; OHT, ocular hypertension.

The responses of ONH astrocytes to glaucoma are also temporally or stage-dependent (Table 3, Figure 4). A moderate and transient elevation of IOP (30 mmHg for 1 h) by anterior chamber cannulation connected to a saline reservoir induced reactive morphological remodeling of mouse ONH astrocytes that peaked at 3 days, including hypertrophy, process retraction, and simplification of cellular shapes (Sun et al., 2013). There were no significant changes in the gene expression profile, no ectopic cell division, no cell death, and no damage to the optic axons. Interestingly, the morphological remodeling was reversible and recovered from 7 days to 6 weeks post-IOP normalization (Sun et al., 2013). A short-term acute elevation IOP (60 mmHg for 8 h) by anterior chamber cannulation in male Brown Norway rats induced transient and reversible ONH astrocyte actin bundle reorientation without axon damage (Tehrani et al., 2016).

**Table 3:** ONH astrocytes changes in glaucoma.

**Species, model, disease stage**	**ONH astrocytes**	**References**
**Mouse**		
C57Bl/6 mice, 3 days-6 weeks after transient OHT	Reversible morphological remodeling, sprouting of longitudinal processes	Sun et al. (2013)
GFAP-GFP transgenic mice, 3 days of OHT	Thinner processes, increased nuclear clusters, reduced AROA	Ling et al. (2020) and Quillen et al. (2020)
DBA/2J mice, 9–16 months	Smaller and retracted processes; reduced spatial coverage, upregulated Galectin-3 expression	Nguyen et al. (2011)
DBA/2J mice, 6 months, no OHT	Sprouting of GFAP-negative processes	Lye-Barthel et al. (2013))
DBA/2J mice, 9–13 months	Diminished ramification, cytoskeletal reorganization, increased vimentin expression; process hypertrophy; upregulated Cx43 expression, formation of glial scars	Bosco et al. (2016) and Cooper et al. (2016), (2018)
**Rat**		
Male BN rats, hours-5 days after transient OHT	Reversible ONH astrocyte actin bundle reorientation	Tehrani et al. (2016)
Female albino Swiss rats, 7 days of OHT	Process withdrawal in dorsal ONH	Dai C. et al. (2012)
Female albino Wistar rats, 10 days of OHT	Cell proliferation; increased vimentin expression	Son et al. (2010)
Male BN rats, 3 days of OHT	Cx43↓, PCNA↑ at MTZ	Johnson et al. (2000)
Male BN rats, 1–2 weeks of OHT	GFAP↓	Johnson et al. (2000)
Male BN rats, 4–5 weeks of OHT	GFAP↑, nuclear pSTAT3 labeling, process reorientation from transverse to longitudinal	Johnson et al. (2000), Tehrani et al. (2014), and Lozano et al. (2019)
Male Long-Evans rats,6 weeks of OHT	Increased inflammatory cytokine and GFAP↑	Wang et al. (2017)
**Monkey**		
Monkey, 2–15 weeks of OHT	Rounding and migration from the core of the cribriform plates	Hernandez and Pena (1997)
Human		
Human, late stage (postmortem)	Prelaminar: Enlarged soma, process hypertrophy, GFAP↑ LC: Round-shape cell body; process loss, GFAP↑ LC and retina: Upregulated Cx43 expression, GFAP↑	Varela and Hernandez (1997), Hernandez et al. (2000), Wang et al. (2002), and Kerr et al. (2011)

AROA, Area of the retina occupied by astrocytes; OHT, Ocular hypertension; BN rat, Brown Norway rats. Up arrow indicates increased expression of indicated proteins, down arrow indicates decreased expression of indicated proteins.

Persistent OHT for 3–10 days can induce morphological remodeling (process retraction) and cell division of ONH astrocytes (Figure 4). Notably, based on these observations, it was proposed that the damage to the RGC axons is not mechanical but is a consequence of localized loss of metabolic support from the ONH astrocytes (Dai et al., 2012). ONH astrocytes in mice with microbeads induced-OHT for 3 days had overall thinner processes, a reduced area fraction of GFAP expression, and an increased number of nuclear clusters (Ling et al., 2020). The junctions between astrocytes and their basement membranes were disrupted, resulting in the reorientation of cell processes, new collagen formation, and cell proliferation after 1 week of OHT in mice (Quillen et al., 2020). Studies using female Albino Swiss rats with anterior chamber magnetic microspheres induced OHT found that withdrawal of astrocytic processes in the dorsal side of the ONH occurs at 7 days of OHT induction, coinciding with the site of initial axon loss. There is no indication of distortion or compression of the axons (Dai et al., 2012). Studies using female Wistar rats with laser induced OHT found that vimentin^+^ astrocytes increased in the post-laminar ONH 10 days after laser treatment, indicating that the astrocyte activation is an early event (Son et al., 2010).

Persistent OHT for 3–14 days can induce morphological remodeling (process retraction and reorientation, loss of gap junction), reduced GFAP expression, and cell division of ONH astrocytes (Figure 4). Studies using male Brown Norway rats with episcleral vein injection of hypertonic saline found that ONH astrocytes were stressed 3–14 days after OHT induction, as evidenced by reduced expression of Cx43 and GFAP and increased expression of PCNA (Johnson et al., 2000). About 2% of ONH cells were divided in this rat model. Rat ONH of ocular hypertension for 5 weeks had more Sox2+ astrocytes than controls, and co-staining of Ki67 and Sox2 proved that 80% (anterior ONH) and 66% (transition zone of ONH) of mitotic cells were astrocytes (Lozano et al., 2019). Reduced gap junction (Cx43 expression), cell division, and decreased GFAP expression are early features of ONH astrocytes, suggesting that ONH astrocytic cytoskeletal reorganization was the early event associated with OHT (Johnson et al., 2000), including the reorientation of ONH astrocyte processes from transverse to longitudinal, as indicated by phalloidin-labeling (Tehrani et al., 2014). GFAP expression increased in ONH astrocytes only at a late stage or with long-term (about 1 month) OHT induction in rats (Johnson et al., 2000).

DBA/2J mouse models of glaucoma are widely used to study ONH astrocytes (Figure 4). ONH astrocytes can phagocytose large axonal evulsions. Some ONH astrocytes at the myelination transition zone (MTZ) express the phagocytosis-related galectin-3 (Gal3). These cells up-regulated Gal3 expression in a γ-synuclein-dependent way in DBA/2J mouse, suggesting that failure to clear axon-derived debris at the MTZ, such as γ-synuclein, may be related to axon loss (Nguyen et al., 2011).

DBA/2J mice also show ONH astrocyte remodeling, including process retraction and sprouting. GFP-labeled astrocytes in DBA/2J mice had thickened and simplified processes. They became smaller and retracted processes, thus having a reduced spatial coverage (Lye-Barthel et al., 2013). DBA/2J mice had diminished ramification and reorientation of astrocyte processes in the early phase of the disease (Cooper et al., 2016, 2018). There was localized sprouting of new processes in the early stages (6 months) of the disease before detectable RGC death. These new processes had no GFAP protein and grew into the axon bundle (Lye-Barthel et al., 2013). In microbeads-induced OHT mice, GFP-labeled MTZ astrocytes extend new processes and invade ONH axon bundles. These longitudinal processes are likely a common feature of glaucomatous ONH astrocytes, as most processes are transverse in naïve ONH astrocytes (Wang et al., 2017). These new longitudinal processes contain sparse intermediate filaments and no subcellular organelles, but some apparently degenerating mitochondria may be of axonal origin (Wang et al., 2017).

In late-stage glaucoma patients, the ONH astrocytes are activated and characterized by morphologic changes (enlarged soma and thicker cellular processes in the prelaminar region, and round-shape cell body and loss of processes in the LC) and increased GFAP expression (Varela and Hernandez, 1997; Hernandez et al., 2000; Wang et al., 2002). The gap junction protein Cx43 also increased in LC and the retina of post-mortem human POAG eyes (Kerr et al., 2011; Figure 4). In a monkey model of glaucoma, ONH astrocytes demonstrate rounding and migration from the core of the cribriform plates as early as 4 weeks after the elevation of IOP (Hernandez and Pena, 1997). Consistent with changes in human glaucoma, ONH astrocytes were also activated in old DBA/2J mice, with increased expression of GFAP and Cx43 (Son et al., 2010; Cooper et al., 2018). The activated astrocytes can form glial scars to cover lesions of axonal degeneration (Bosco et al., 2016).

### Visual brain astrocytes in glaucoma

Most studies regarding astrocytes in the visual brain were performed in the monkey glaucoma model. In monkeys with laser-induced OHT for 2–15 weeks, GFAP expression increased in the lateral geniculate nucleus (LGN), especially in the layers receiving a neuronal input from the high IOP eye (Sasaoka et al., 2008). GFAP expression increased in the LGN and the visual cortex (V1), accompanied by loss of neuronal metabolic activity as assessed with cytochrome oxidase (CO) histochemistry (Lam et al., 2009). Reactive astrogliosis occurred in the magnocellular and parvocellular LGN layers of monkeys with unilateral glaucoma. A quantitative study indicated a linear relationship between increased GFAP expression and ONH axon loss (Dai Y. et al., 2012). LGN astrocyte activation in the monkey glaucoma model can also be detected by PET imaging with [^11^C] PK11195, a PET ligand for peripheral-type benzodiazepine receptor (PBR). These data suggested that activated glial markers such as PBR in the LGN may be useful noninvasive biomarkers for diagnosing glaucoma (Shimazawa et al., 2012).

In the superior colliculi (SCs) of DBA/2J mouse, loss of anterograde transport of RGC axon was accompanied by hypertrophy of brain-derived neurotrophic factor (BDNF)-expressing astrocytes, which might represent an intrinsic mechanism to mitigate the effects of RGC axon damage (Crish et al., 2013). In male Long-Evans rats with unilateral OHT induced by EVC, pro-inflammatory markers (such as IL-1β and TNFα) and astrocyte activation were identified in hypertensive and normotensive RGC projection sites in the SCs. These results suggested a complicated role of the SCs in the propagation of neuroinflammatory events induced by unilateral OHT (Sapienza et al., 2016). In a ferret model of OHT, loss of neurons and activation of astrocytes occurred in the LGN layers receiving projections from OHT eyes especially in the C layer (Fujishiro et al., 2020).

In summary, the response of astrocytes to glaucoma is quite heterogeneous and is stage, region, and spatially dependent. In the early stage of glaucoma, cell density and GFAP expression of retinal astrocytes are reduced, and the morphological remodeling of ONH astrocytes is reversible. Reduced Cx43 and GFAP expression and cell division are early features of ONH astrocytes. Longitudinal processes with degenerating mitochondria are a common feature of glaucomatous ONH astrocytes. Withdrawal of ONH astrocytic processes may result in localized loss of metabolic support from the ONH astrocytes to RGC axons. The astrocytes in the visual brain also respond to glaucoma insult. Understanding the molecular mechanism of these responses is necessary to develop an effective therapy for glaucoma in the future.

## Heterogeneous Astrocyte Function and Neuronal Degeneration in Glaucoma

Neuroinflammation and mitochondrial dysfunction are the most important mechanisms underlying RGC death and axon degeneration in glaucoma (Ahmed et al., 2004; Yang et al., 2007; Williams et al., 2017; Tribble et al., 2021). However, it is difficult to distinguish the contributions of astrocytes from those of microglia and Müller glia because they often become reactive in concert and function in coordination as one unit (Liddelow and Barres, 2017). Thus, transgenic or knockout mouse models have important roles in determining the unique functions of astrocyte reactivity in glaucoma (Table 4). As stated above, these functions are heterogeneous, some are beneficial, but some are detrimental to RGCs in glaucoma.

**Table 4:** Gene knockout studies in glaucoma or IR mouse models.

**Mouse models**	**Knockout or mutated genes**	**Effects on RGCs**	**References**
DBA/2J mice	Germline, C3	RGC loss↑	Harder et al. (2017)
DBA/2J mice	Germline, C3ar1	RGC loss↓	Harder et al. (2020)
DBA/2J mice	C1qa mutation	RGC loss↓	Howell et al. (2011)
OHT mice	Astrocytes specific, IκKβ or p65	RGC loss↓	Yang et al. (2020)
OHT mice	Germline, IL-1α, TNFα, and C1q	RGC loss↓	Guttenplan et al. (2020)
OHT mice	Astrocytes specific, Caspase 8	RGC loss↓	Yang et al. (2021)
OHT mice	Astrocytes specific, Stat 3	RGC loss↑	Sun et al. (2017)
OHT mice	Astrocytes specific, Cx43	RGC loss↑	Cooper et al. (2020)
IR mice	Astrocytes specific, Cx43	RGC loss↓	Toychiev et al. (2021)
OHT mice	Germline, C36	RGC loss↓	Akopian et al. (2017)
OHT mice	Astrocytes specific, Ucp2	RGC loss↓	Hass and Barnstable (2019)

IR, Ischemia-reperfusion retinal injury; OHT, Ocular hypertension. Up arrow indicates increased RGC loss, down arrow indicates decreased RGC loss.

### Complement C3 and EGFR signaling in astrocytes are beneficial to RGCs in glaucoma

The ONH astrocytes exhibit increased complement C3 expression in DBA/2J mice, without detectable RGC dysfunction and axon degeneration (Harder et al., 2017). Knocking out the C3 gene significantly increased the number of DBA/2J eyes with nerve damage and RGC loss after IOP elevation. The C3-dependent astrocytic response involves EGFR signaling. EGFR inhibitor AG1478 substantially increased the number of eyes with moderate or severe glaucoma. Thus, in the mouse model of glaucoma, ONH astrocytes produce C3 and activate EGFR signaling to support RGC survival (Harder et al., 2017; Figure 2). This result is consistent with observations that patients with primary angle-closure glaucoma (PACG) or POAG have lower plasma C3 levels, which were negatively associated with the disease severity (Li et al., 2017, 2018).

However, inhibition of C3 activation in the retina of DBA/2J mice, by intravitreal injections of AAV2.CR2-Crry, can suppress C3d deposition in RGCs, which results in significant long-term neuroprotection of ONs and the retina. Thus, it is possible that there are compensatory changes following C3 knockout that do not occur with local inhibition of C3 activation (Bosco et al., 2018).

Similarly, the C3 cleavage product C3a can recruit microglia and infiltrating monocytes that express C3a receptor-1 (C3ar1), but C3ar1 is a detrimental neuroinflammatory factor in DBA/2J glaucoma, as C3ar1 knockout lowered the risk for RGC degeneration (Harder et al., 2020). Indeed, C3a/C3 ratio increases in aqueous humor (AH) and serum in progressive POAG patients but not in stable POAG patients (Hubens et al., 2021). In contrast to C3 in astrocytes, C1q upregulation in microglial cells is also an early response to IOP elevation but is detrimental to RGCs as described below (Howell et al., 2011; Guttenplan et al., 2020).

### NF-κB signaling in astrocytes is detrimental to RGCs in glaucoma

In the CNS, A1 neurotoxic astrocytes are activated by the NF-κB signaling pathway (Lian et al., 2015). In human donor eyes with glaucoma (Yang et al., 2011) and glaucoma rat model (Tezel et al., 2012), astrocyte-specific nuclear factor-kappaB (NF-κB) is critical for the transcriptional regulation of neuroinflammation (Figure 2). NF-κB represents a family of transcription factors, including NF-κB1, NF-κB2, RelA (also named p65), RelB, and c-Rel. The NF-κB proteins are usually sequestered in the cytoplasm by IκB kinases. Thus, the transcriptional activity of NF-κB depends on the degradation of IκB *via* a process that requires specific kinases (IκK). The IκK-subunit β (IκKβ) is the central activating kinase involved in IκB degradation. GFAP-Cre/ERT2 induced astrocyte-specific knockout of IκKβ or p65 gene reduced pro-inflammatory cytokines (such as TNFα, IFNγ, IL1, and IL2) in the retina and optic nerve of OHT mice. Inhibition of astroglial NF-κB signaling reduced RGC death and improved the retinal function of OHT mice. Interestingly, IκKβ or p65 deletion in astrocytes also reduced pro-inflammatory cytokine production in microglia (Yang et al., 2020).

### Astrocytes activated by IL-1α, TNFα, and C1q are detrimental to RGCs in glaucoma

In the CNS, activated microglia-derived cytokines, including IL-1α, TNFα, and C1q, activate A1 neurotoxic astrocytes (Liddelow et al., 2017). DBA/2J mice with C1qa gene mutation were protected from RGC degeneration (Howell et al., 2011). Triple knockout IL-1α, TNFα, and C1q genes reduced reactive astrogliosis and protected RGCs (especially SMI-32^+^ α-RGCs) from microbeads-induced chronic OHT (Guttenplan et al., 2020; Sterling et al., 2020). Interestingly, subcutaneous injections of NLY01, a long-acting GLP-1R (glucagon-like peptide 1 receptor) agonist, can reduce microglia production of C1q, TNF-α, and IL-1α and RGC loss in this OHT model (Sterling et al., 2020). Ceramides can induce RGC cell death by inducing TNF-α secretion from optic nerve head astrocytes (Fan et al., 2021).

Because the C1qa gene mutation and triple knockout are all germline and not astrocyte-specific, if these protective effects are solely related to the reduced reactive astrogliosis is unknown. Germline knockout of these cytokine genes can cause changes in other cell types, for example, RGCs, Müller glia, and microglia, which may also contribute to these observed protective effects in OHT mice.

### Caspase-8 in astrocytes induces inflammation and is detrimental to RGCs in glaucoma

Caspase-8 is the initiator caspase of the TNFR-mediated extrinsic apoptosis. It inhibits necroptosis mediated by RIPK3 and MLKL (Fritsch et al., 2019), and promotes NF-κB-mediated cell survival and inflammation (Su et al., 2005). Caspase-8 is detected in the apoptotic RGCs in human and animal models of glaucoma (Yang et al., 2011; Chi et al., 2014). GFAP-Cre/ERT2 induced astrocyte-specific knockout of caspase-8 suppressed neurodegenerative inflammation and protected RGC structure and function in microbeads-induced OHT eyes (Yang et al., 2021; Figure 2).

### STAT3 in astrocytes is beneficial to RGCs in glaucoma

The astrocytes exhibit increased STAT3 expression in human glaucoma (Yang et al., 2011) and animal glaucoma models (Johnson et al., 2011; Zhang et al., 2013; Lozano et al., 2019). STAT3 is a member of the Jak-STAT signaling pathway and is activated by phosphorylation through cytokines and growth factors. STAT3 is crucial for A2 reactive astrogliosis (Herrmann et al., 2008; Zhang et al., 2013) and mediates the neuroprotective functions of reactive astrocytes (Justicia et al., 2000; Yamauchi et al., 2006; Zhang et al., 2013; Ben Haim et al., 2015; Sun et al., 2017). Indeed, in transient and chronic OHT mice, GFAP-Cre induced astrocyte-specific knockout of STAT3 impaired astrogliosis (loss of the large distinct glial tubes and the overall honeycomb arrangement, hypertrophy of astrocyte processes) in the ONH, and exacerbated RGC injury (losing 20%–30% more RGCs compared with control) and reduction in the positive scotopic threshold response (Sun et al., 2017; Figure 2). Similar results are also observed using the retrobulbar injection of STAT3 inhibitor to rats with transient OHT (Wong et al., 2015). Thus, early reactive astrocyte remodeling in glaucoma is adaptive and beneficial and is essential for the survival of RGCs.

### Gap junction among astrocytes is beneficial to RGCs in glaucoma

Astrocytic processes are organized in parallel and share cytoplasmic information *via* communication through gap junctions (GJs) comprised primarily of connexin 43 (Cx43). Gap junctions also allow functional coordination among the members of the neurovascular unit, such as glial cells, endothelial cells, pericytes, and neurons. Astrocytes create and store glycogen as a safeguard against stress. They provide nutritional supplements and energy resources for surrounding neurons (Vecino et al., 2016). This metabolic collaboration between astrocytes and neurons is important to tissue survival during injury. Cx43 expression decreases during the first 2 weeks after OHT induction (Johnson et al., 2000), but increases at the late stage of human glaucoma (Kerr et al., 2011), and DBA/2J mouse model of glaucoma (Son et al., 2010; Cooper et al., 2018). There is evidence that elevated IOP activates C3 and EGFR signaling, which is beneficial to RGCs at the early stage of the disease (Harder et al., 2017). However, EGFR signaling can phosphorylate Cx43 and shut down gap junctions, thus disrupting the intercellular communication between astrocytes (Malone et al., 2007). So, both early and late stages of glaucoma may suffer from the reduced function of the gap junction.

GFAP-Cre-ERT2 induced astrocyte-specific knockout of Cx43 gene (Cx43 CKO) in the retina, optic nerve, and superior colliculi (SCs). These Cx43 CKO mice proved that astrocytes in the OHT-stressed ONH can receive glycogen from other astrocytes in the un-stressed contralateral ONH *via* gap junction. Thus, Cx43 CKO eyes lost visual function much faster than controls upon unilateral IOP elevation. These results demonstrate that glucose and its metabolites are redistributed from healthy to stressed ONH through an astrocyte Cx43-mediated network within the optic projection (Cooper et al., 2020). However, GFAP-Cre induced Cx43 knockout indicated that gap junctional coupling between retinal astrocytes exacerbates RGCs loss in ischemia-reperfusion retinal injury (Toychiev et al., 2021). In addition, in contrast to these beneficial effects of astrocyte gap junction in glaucoma, neuronal gap junctions are detrimental to RGCs in glaucoma, as pharmacological blockade of gap junctions or knock out connexin 36 (Cx36) subunits, which are highly expressed in retinal neurons, protected RGCs and optic nerve axons in a mouse model of glaucoma (Akopian et al., 2017). The possible reason is that gap junctions can form conduits through which toxic molecules from dying cells pass to and harm coupled neighbor neurons.

### Astrocyte mitophagy is beneficial to RGCs in glaucoma

In animal models of glaucoma, the mitochondria of many cell types (such as astrocytes, Müller glia, and RGCs) become dysfunctional, resulting in energetic deficits, increased ROS, and calcium imbalance. Mitophagy can remove damaged mitochondria and is the major mechanism for mitochondrial quality control. Impaired mitophagy can cause neurodegenerative diseases such as Parkinson’s disease (Geisler et al., 2010; Vives-Bauza et al., 2010). Mitochondrial uncoupling proteins (Ucp) can uncouple the electron transport chain from ATP synthase activity and decrease membrane potential (Ψm). The mitochondrial uncoupling protein 2 gene (Ucp2) is expressed in the retina, can decrease electron transport chain efficiency and ROS level, and increase energy expenditure (Arsenijevic et al., 2000). GFAP-Cre-ERT2 induced astrocyte-specific knockout of Ucp2 gene, increased levels of mitophagy in the retina, decreased oxidative protein modification, and reduced RGC death in microbeads-induced OHT mice. Ucp2 deletion facilitates increased mitochondrial function by improving quality control (Hass and Barnstable, 2019; Figure 2). These results are consistent with findings that overexpression of the E3 ubiquitin ligase parkin, or optic atrophy type 1 (OPA1) can restore dysfunctional mitophagy in OHT rats and increase RGC survival (Dai et al., 2018; Hu et al., 2018). Astrocytes can internalize damaged RGC mitochondria (Davis et al., 2014). Ucp2 knockout, which enhances glial mitophagy, may also enhance trans-cellular degradation of damaged RGC mitochondria or other components to protect against mitochondrial damage in glaucoma (Hass and Barnstable, 2019).

In summary, these studies suggested that astrocytes have both beneficial and detrimental functions to RGCs in glaucoma. They can regulate neuroinflammation, mitochondrial dysfunction, and energy redistribution in glaucoma (Table 4).

## Conclusion and Remarks

Glaucoma has multiple triggers that involve multiple cell types (RGCs, glial cells) and anatomic locations along the visual pathway. Multiple molecular pathways with biomechanical, vascular, metabolic, oxidative, and inflammatory elements are likely engaged with the degeneration of RGCs in glaucoma. In this review, we demonstrated that astrocytes are a heterogeneous population of cells in the retina, ONH and visual brain and play multiple roles in maintaining homeostasis and regulating neuronal activities. These astrocytes also have very heterogeneous reactions to local insults. Similarly the response of astrocytes to glaucoma is also heterogeneous and is stage, region and spatially dependent. We also discussed their contributions to glaucoma pathologies, especially neuroinflammation and mitochondrial dysfunction, as demonstrated by several gene knockout studies. Thus, therapeutic strategies targeting the heterogeneity of astrocytes by enhancing their beneficial reactivity or suppressing their detrimental reactivity offer alternative options for glaucoma treatment in addition to IOP control. For instance, overexpression of Stat3 or C3, knockdown of IκKβ/p65, caspase 8 and mitochondrial uncoupling proteins (Ucp2) in astrocytes may reduce ganglion cell loss in glaucoma patients in the future. Astrocytes-targeting treatment will be an important option in the management of glaucoma. This conclusion requires not only a thorough understanding of the distinct molecular mechanisms of heterogeneous reactivity of astrocytes in glaucoma but also carefully designed clinical trials.

## Author Contributions

YT, YC, and DC conceived and designed the manuscript, wrote, edited, and approved the manuscript. All authors contributed to the article and approved the submitted version.

## Funding

This study was supported by grants to DC from the National Natural Science Foundation of China (81870665, 82171063).
